# Supplementary material for: Characterization of heart rate variability in end-stage renal disease patients after kidney transplantation with recurrence quantification analysis
Source: PLoS One. 2024 May 1;19(5):e0299156. doi: 10.1371/journal.pone.0299156 (PMC11062555; doi:10.1371/journal.pone.0299156)
Supplement: S1 File — (DOCX) [file pone.0299156.s001.docx]

**List of abbreviations**

| BMI: Body mass index |
| --- |
| BUN: blood urea nitrogen |
| CKD: Chronic Kidney Disease |
| CKD-EPI: Chronic Kidney Disease Epidemiology Collaboration |
| ECG: Electrocardiogram |
| ESRD: End-stage renal disease |
| HF: High frequency |
| HRV: Heart rate variability |
|  |
| LF: Low frequency |
| meanNN: mean of NN intervals |
| pNN20: percentage of consecutive NN intervals that differ in more tan 20 ms |
| RQA: Recurrence quantification analysis |
| SDNN: standard deviation of NN intervals |
| SDSD: standard deviation of the difference between consecutive NN intervals |
| T1: Recurrence time of the 1st type |
| T2: Recurrence time of the 2nd type |
|  |
|  |
|  |
|  |
