# Supplementary material for: Characterization of heart rate variability in end-stage renal disease patients after kidney transplantation with recurrence quantification analysis
Source: PLoS One. 2024 May 1;19(5):e0299156. doi: 10.1371/journal.pone.0299156 (PMC11062555; doi:10.1371/journal.pone.0299156)
Supplement: Attachment — Submitted filename: Response to Reviewers - R2.pdf [file pone.0299156.s005.pdf]

Manuscript PONE-D-23-02358.R1 "Characterization of nonlinear heart rate variability of end-stage renal disease patients after kidney transplantation"

Response to reviewers

We appreciated all the observations and questions made to our manuscript, all of which we are sure will improve our paper's quality. Next, we will provide answers to the questions and observations made by the reviewers.

Reviewer #5

Comment: The authors set out to assess the response of heart rate variability (HRV) linear indices and recurrence plot quantitative analysis (RQA) measures in an active standing test on kidney transplant recipients. They concluded that HRV of kidney recipients shows a diminished variability during active standing, in comparison with supine position. That the dynamic behavior of HRV also changed in response to active standing with a consistent predominance of vertical structures. They went on to suggest that these changes suggest a marked sympathetic response to active standing, and a preserved capacity to respond to such orthostatic challenges with appropriate adjustments in the dynamic behavior of the cardiovascular control system.

This is an interesting topic that definitely requires more study and I am appreciative of these authors efforts. The paper has a number of deficiencies:

1. It is hard to read. The writing in English could be better, shorter and more succinct. For instance, the first sentence either needs to be two sentences or have a semi-colon. "Heart rate variability (HRV) is the instantaneous change of heart rate, these variations of heart rate are tightly related to respiratory and autonomic modulation of the cardiovascular system." Consider splitting this into two sentences or use a semi-colon instead of the coma. The sentence " However a kidney transplant is the treatment of choice for ESRD over lifetime dialysis in most clinical scenarios" is also unnecessary since it is universally expected that kidney transplantation is the best treatment modality for patients with ESKD.

Response: Thank you because of the observation. We reviewed all the text and made changes to improve the writing in English, as you will see in different sections of the paper. We agree with you regarding kidney transplant as the universally accepted best treatment choice in patients with end-stage renal disease. We removed the sentence.

Comment: 2. The authors suggest that kidney transplantation may be the cause of the "diminished variability during active standing, in comparison with supine position." This however is an observational study and we cannot draw causality from the results of this study.

Response: We completely agree with you. Our study cannot establish causality in the improvement of heart rate variability resulting from kidney transplantation.

As we mention on page 12, paragraph 2: "…our study does not have HRV measurements of these patients before kidney transplantation; hence, it cannot be determined whether kidney transplantation improves RQA measures". In the same paragraph, we compare the present results with previous work on patients treated with hemodialysis. At the end, we mention that: "A better eGFR may be linked to an improvement in HRV measures as

observed in our work”. However, we recognize that further studies are required to assess if this relationship is related to the improvement of cardiovascular function due to kidney transplantation.

To avoid confusion in our Conclusions section, we included a paragraph emphasizing that further studies are required to demonstrate causality.

Comment: 3. The study population is small and young. The study population has only 16 patients and the average age was 32 years. The average age for patients with ESRD is much higher (45-60s when initiating dialysis in Mexico) and it would have been more interesting and applicable if the authors studied patients with reflect the general ESRD population. To be fair, other studies on HRV also had younger populations (e.g. Martín Calderón-Juárez et al's paper had average age of 27+/- 8 years). In general, the paper's generalizability is limited.

Response: We agree that the patients included in our study are very young, and we understand that this situation may be representative of most patients undergoing kidney transplantation in your country, with a mean age of 29 years. However, we also acknowledge that national statistics can vary, and the global reality may present a different situation regarding the age of patients with end-stage chronic kidney disease. Our study expanded the discussion to address this point and provide a more comprehensive understanding of the situation.

Reviewer #6

Comment: Thank you for your manuscript.

As a nephrology reviewer I am unable to understand the multiple abbreviations. Not only that what these abbreviations stand for and their significance.

Response: The most recent manuscript includes a list of abbreviations.

Comment: The authors need to clarify the following statements.

1.What do you mean ESRD with Kidney transplantation? Is this a case of allograft failure?

Response: We agree with you; it can be confusing. The patients included in the study had evidently experienced end-stage renal disease and received dialysis therapy at some point. At the time of their inclusion in the study, they had a functional kidney transplant. The sentence has been modified to avoid confusion (page 4, line 1).

Comment: 2.What is the implications of replacing heart beats in the final analysis. Is 5% accepted standards?

Response: According to the guidelines for heart rate variability analysis (Reference 1), the analysis should involve only RR intervals measured during sinus rhythm only. Therefore, RR intervals that involve extrasystoles should not be included. The guidelines indicate that up to 5% of replaced heartbeats are acceptable. The reference to guidelines is now on page 6, paragraph 1.

Comment: 3.What is the final message from the study and is there anything new?

Response: As mentioned in the Conclusions section, our work demonstrated that kidney recipients show a preserved physiological response to an active orthostatic challenge. The response is consistent with increased sympathetic activity during orthostasis. Moreover, the recurrence plot analysis also evidenced interesting changes in the dynamic behavior of HRV.

Comment: 4. It would be instructive to know what anti-hypertensives that the transplant recipients were on and their impact on HRV.

Response: Indeed. We included that information in the article (page 4, paragraph 1). Only three patients were on antihypertensive medications, of whom only one was taking a low-dose non-cardioselective beta-blocker.

Comment: Improvement in autonomic activity with improvement of eGFR is a known phenomenon.

Response: Correct. However, the study of autonomic activity is not straightforward. Here we used a non-invasive approach with heart rate variability during a controlled physiological maneuver (active standing). The response of the cardiac autonomic modulation to this maneuver is well known for healthy and end-stage renal patients treated by hemodialysis. Our work shows that kidney transplant recipients have a preserved autonomic response to such a maneuver. Also, nonlinear indices of heart rate variability have emerged as a helpful tool to analyze this, and that is what we explored in this study. Our results showed that the correlations between eGFR and heart rate variability indices were more evident for most indices when the physiological stress of active standing was applied.
