# Supplementary material for: Characterization of heart rate variability in end-stage renal disease patients after kidney transplantation with recurrence quantification analysis
Source: PLoS One. 2024 May 1;19(5):e0299156. doi: 10.1371/journal.pone.0299156 (PMC11062555; doi:10.1371/journal.pone.0299156)
Supplement: Attachment — Submitted filename: Rebutal letter_09.21.2023.pdf [file pone.0299156.s006.pdf]

Characterization of heart rate variability in end-stage renal disease patients after kidney transplantation with recurrence quantification analysis

Response to reviewers

Reviewer #6: The authors have addressed the comments raised by the reviewers.

Only concern is that the manuscript has many technical issues which are beyond the scope of transplant physicians. This manuscript is written for the cardiology sub-speciality audience.

For a nephrologist the fact that that the autonomic dysfunction improves post transplantation is well known.

It would have been more instructive if an attempt was made if the changes are merely attributed to improvement of GFR or other factors like improving anemia, PTH levels, electrolytes etc are also responsible.

Response: We thank the reviewer for their comments, which contributed to the quality improvement of the present manuscript. We acknowledge that the complex physiology behind eGFR and HRV might be influenced by a large and intricated number factors. The exploratory work that we present will contribute as a precedent to propose further research to help elucidate the mechanism and clinical implications of the correlation between HRV and eGFR. We address this issue in the revised version of the manuscript (Discussion section, page 12, paragraph 1, lines 11- 18).
