# Supplementary material for: Characterization of heart rate variability in end-stage renal disease patients after kidney transplantation with recurrence quantification analysis
Source: PLoS One. 2024 May 1;19(5):e0299156. doi: 10.1371/journal.pone.0299156 (PMC11062555; doi:10.1371/journal.pone.0299156)
Supplement: Attachment — Submitted filename: RebuttalLetterSubmit.pdf [file pone.0299156.s007.pdf]

Manuscript PONE-D-23-02358R3 “Characterization of heart rate variability in end-stage renal disease patients after kidney transplantation with recurrence quantification analysis”

Response to Editor and Reviewers

Comment from the Editor (Journal Requirements)

Please review your reference list to ensure that it is complete and correct. If you have cited papers that have been retracted, please include the rationale for doing so in the manuscript text, or remove these references and replace them with relevant current references. Any changes to the reference list should be mentioned in the rebuttal letter that accompanies your revised manuscript. If you need to cite a retracted article, indicate the article’s retracted status in the References list and also include a citation and full reference for the retraction notice.

Response: Thank you for this indication. We reviewed the reference list to verify that is complete and correct. We found that only one reference (number 25) had been retracted recently (November 2023):

25. Shi W, Zhang J, Chen D, Chen X, Duan W, Zhang H. Heart Rate Variability and Chronic Kidney Disease in Patients with Type 2 Diabetes. Appl Bionics Biomech. 2022;2022:2475750. Epub 20220517. doi:10.1155/2022/2475750. PubMed PMID: 35619730; PubMed Central PMCID: PMCPMC9129959.

We eliminated both the reference and the following phrase from page 12, paragraph 1: “In another study in which HRV was evaluated within 24 hours recordings, the correlation between SDNN and eGFR in patients with chronic kidney disease and type 2 diabetes was not significant [25].”

We also updated the order for the following references:

23. García-García G, García-Bejarano H, Breien-Coronado H, Perez-Cortez G, Pazarin-Villaseñor L, de la Torre-Campos L, et al. Chapter 9 - End-Stage Renal Disease in Mexico1. In: García-García G, Agodoa LY, Norris KC, editors. Chronic Kidney Disease in Disadvantaged Populations: Academic Press; 2017. p. 77-83.

24. Lerma C, Minzoni A, Infante O, Jose MV. A mathematical analysis for the cardiovascular control adaptations in chronic renal failure. Artif Organs. 2004;28(4):398-409. Epub 2004/04/16. doi: 10.1111/j.1525-1594.2004.47162.x. PubMed PMID: 15084202.

25. Tahrani AA, Dubb K, Raymond NT, Begum S, Altaf QA, Sadiqi H, et al. Cardiac autonomic neuropathy predicts renal function decline in patients with type 2 diabetes: a cohort study. Diabetologia. 2014;57(6):1249-56. doi: 10.1007/s00125-014-3211-2.

Comments from the Reviewers

6. Review Comments to the Author

Please use the space provided to explain your answers to the questions above. You may also include additional comments for the author, including concerns about dual publication, research ethics, or publication ethics. (Please upload your review as an attachment if it exceeds 20,000 characters)

Reviewer #1: The manuscript has been improved. The authors replied satisfactorily to all my issues and took into account the suggestions given. I have no additional comments.

Reviewer #6: The revised manuscript attempts to address most of the issues.

The central concern about the relevance to the nephrology community still remains.

Response: We addressed previously this concern, specifically about the correlation between eGFR and HRV indices. As we mentioned in the previous round, such correlation is already known among nephrologists, as is cited in the manuscript (page 12, lines 1 to 3): "Correlation between greater HRV and better eGFR has been observed in patients with chronic kidney disease in a broad population with different autonomic maneuvers [25] ."

Moreover, as we mentioned in the previous round, the factors that may explain such correlation are diverse and not fully elucidated. In our manuscript, we discuss some of these clinically relevant factors (page 12, lines 11 to 18). Furthermore, as we mention in the same paragraph: "In this exploratory study, such factors were not evaluated, and further research is needed to assess their specific role with HRV modulation and eGFR."

We have added the following sentence in the same paragraph to point out the relevance of our findings:

"Since our approach of non-linear HRV indices obtained by recurrence plot analysis during a physiological stimulus (active standing) has been useful to address the dynamic adaptability in ESRD patients treated by HD [5,7], the current findings of the correlation between eGFR and RQA measures could motivate future investigation about the adaptability of the cardiovascular control mechanisms in transplanted patients."

7. PLOS authors have the option to publish the peer-review history of their article (what does this mean?). If published, this will include your full peer review and any attached files.

If you choose "no", your identity will remain anonymous but your review may still be made public.

Response: Yes
