# Supplementary material for: Characterization of heart rate variability in end-stage renal disease patients after kidney transplantation with recurrence quantification analysis
Source: PLoS One. 2024 May 1;19(5):e0299156. doi: 10.1371/journal.pone.0299156 (PMC11062555; doi:10.1371/journal.pone.0299156)
Supplement: Attachment — Submitted filename: Characterization of nonlinear heart rate variability of end.docx [file pone.0299156.s003.docx]

This paper concerns “Characterization of nonlinear heart rate variability of end-stage renal disease patients after kidney transplantation”

It is a struggle for me to read the paper because of many language issues and a huge number of abbreviations. I am not familiar with all these cardiology terms and abbreviations, so that does not help. Although the subject is interesting, after trying to read it several times, at present I cannot comment properly because of the numerous textual issues.

Table 2 abbreviations after normal text would make it more readable.

A list of abbreviations is also required

A clear legend of the figures is necessary

The following sentences need improvement.

1. However, it is unknown whether this response is recovered after definitive treatment with kidney transplantation.
2. We studied HRV dynamics by obtaining short-term ECG recordings from kidney transplant recipients that underwent active standing stimulation
3. Larger estimated glomerular filtration rate (eGFR) in significantly correlated with

broader gross HRV in supine position and during active standing. The loss in HRV during active standing may indicate a preserved sympathetic response to external stimul

4. is known that chronic sympathetic hyperactivity produces decreased HRV modulation with predominance of lowfrequency oscillations, and a blunted HRV response to active standing
5. This tool allows to graphically represent the recurrence of a system to a particular dynamical state
6. This maneuver allows studying of the effects of volume redistribution and sympathetic

stimulation of the cardiovascular system

7. However a kidney transplant is the treatment of choice for ESRD
8. We included 16 patients with ESRD after kidney transplantation that were recruited during followup consultation in a second-level hospital in Mexico with a median age of 32 27-35 years old
9. and triple immunosuppression treatment with a calcineurin inhibitor plus prednisone and mycophenolate.
10. Subjects were asked to avoid caffeinated beverages and other stimulants
11. We name the final HRV time series as NN
12. We calculate the mean of NN time series (meanNN
13. Embedding dimension was calculated at the first local minimum reaching zero in the fixed amount of neighbors function
14. Categorical variables are reported as absolute frequency (relative frequency) and continuous

variables as median (interquartile range).

15. Time domain indices, meanNN, SDNN, SDSD, and pNN20 are larger during supine position in comparison to active standing
16. Few correlations are observed in supine position (SDSD, pNN20, DET and ENT), but during active standing a significant correlation is found between eGFR and most of HRVmeasures (SDNN, SDSD, Pnn20, LF, HF, LF/HF, DET, LAM, TT and T2).
17. Also, a larger standard deviation of 5-minutes intervals in 24 hours (SDANN) of kidney transplant recipients without diabetes than those recipients without diabetes
18. Here, patients after kidney transplantation
19. However, we found here that in transplant recipients, these measures increase their value during active standing.
20. change in healthy subjects in response to active standing. given that the rapid changes in heart rate
21. is due to dysautonomia. This
22. other experimental scenarios [14, 18], which are not found in patients with ESRD but after HD treatment.
23. approach such be conducted
24. our study do not have
25. it is likely that the changes in RQA measures in active standing (e.g. DET, ENT, LAM and TT) may be a result of fluid overload
26. resistance [5, 25], these
27. As we show in this
28. is related to better eGFR
29. to a improve in HRV
30. to explore this relation and determine
31. in comparison with supine position
32. a consistent predominance of vertical structures (LAM and TT).
33. The larger HRV statistical dispersion,
34. by better renal functioning (eGFR).
