# Supplementary material for: Characterization of heart rate variability in end-stage renal disease patients after kidney transplantation with recurrence quantification analysis
Source: PLoS One. 2024 May 1;19(5):e0299156. doi: 10.1371/journal.pone.0299156 (PMC11062555; doi:10.1371/journal.pone.0299156)
Supplement: Attachment — Submitted filename: Reviewers responses.R1submit.docx [file pone.0299156.s004.docx]

**Manuscript PONE-D-23-02358 “Characterization of nonlinear heart rate variability of end-stage renal disease patients after kidney transplantation”**

Response to reviewers

**Reviewer #1**

**Comment:** The study exploits recurrence quantification analysis (RQA) to characterize heart rate variability (HRV) of end-stage renal disease (ESRD) patients after kidney transplantation (KT).
The study is interesting and originally discuss HRV during orthostatic stimulus in ESRD patients after KT. However, some issues need a more profound discussion.
1) The major finding of the present study is the greater regularity of HRV during standing in ESRD patients after KT and this result supports the general improvement after KT. A greater regularity of HRV is compatible with an increased sympathetic modulation during orthostatic challenge as proven in A. Porta et al, J Appl Physiol, 103, 1143-1149, 2007. The link between loss of HRV complexity and postural challenge should be more deeply discussed and corroborated with adequate references.

**Response:** We thank the reviewer for the comments, which greatly help to improve the clarity and quality of our work. In the revised version of the manuscript, we discuss in greater detail the effects of active standing (page 10).

**Comment:** 2) It remains unclear why the authors used RQA instead of simpler nonlinear, and more widely utilized, descriptors of HRV irregularity such as markers based on conditional entropy.

**Response:** In an effort to broaden the scope of tools for HRV analysis, we explore the changes of HRV using recurrence plots, as it has been also proposed by other authors (10.1016/j.bspc.2015.10.007, 10.3389/fphys.2020.00040, 10.1371/journal.pone.0249504). However, in the new version of the manuscript we discuss the possibility of using other nonlinear methods, which may be or not simpler (page 12).

**Comment:** 3) The authors evoked the presence of nonlinear dynamics in HRV (e.g., in the title) but no tests were utilized to check the actual presence of nonlinear dynamics. Tests should be carried out to corroborate the choice of a nonlinear marker (see e.g., A. Porta et al, Front Physiol, 6, 71, 2015 for a procedure to test the presence of nonlinear dynamics). In alternative, linear markers of complexity are present (see A. Porta et al, IEEE Trans Biomed Eng, 64, 1287-1296, 2017).

**Response:** In the new version of the manuscript, we changed the title and abstract to fit the purpose of this study, which is to describe HRV by traditional and RQA measures in kidney transplantation, rather than the direct demonstration of a particular dynamical behavior through surrogate data testing.

We acknowledge the usefulness of these tests, and we propose to test nonlinear behavior per se in a larger sample and make direct statistical test against HRV before kidney transplantation (Discussion section page 12).

**Comment:** 4) More details should be given to better understand how the parameters of RQA were chosen. For example, it is unclear how the embedding dimension and the tolerance to assess pattern similarity were selected. Please clarify in the Methods.

**Response:** In the revised version, we explain with more detail the selection of parameters (Methods section page 6), which is based on methodological research in recurrence plots (Marwan et al. 2007, reference 11).

First, we obtain the averaged histogram based mutual information of a given heart rate variability (HRV) time series, from which we obtain the first local minimum of the function. The delay at this local minimum corresponds to the time delay of the signal (tau). After a value for tau is chosen, we use it to compute the false nearest neighbors’ function to determine the dimension (m) for the recurrence plot construction. For the latest, we also take the first local minimum of the function (we provided an overview of this selection in a previous work ̶ (reference 12).

**Comment:** 5) The white squares in the recurrence plots in Fig.1 denote the presence of nonstationarities. Their presence is visible as changes of the mean in the same figure. Since stationarity is a prerequisite of RQA, it should be checked (see V. Magagnin et al, Physiol Meas, 32, 1775-1786, 2011 for a stationarity test). In alternative, the authors should discuss the impact of nonstationarities over the final conclusion of the study.

**Response:** Indeed, it is qualitatively observed the nonstationary behavior of HRV. In a previous work (reference 15), we showed that RQA obtains pertinent information contained in HRV of healthy and ESRD patients, despite its nonstationary behavior (tested by the proposed algorithm). We added in the discussion the potential impact of nonstationarity in our results (page 12).

**Comment:** 6) English language should be profoundly revised (e.g., the second phrase from the bottom of the Abstract). Construction of the phrases should be checked (e.g., the first phrase of the Introduction) as well as the usage of punctuation.

**Response:** We reviewed thoroughly the language usage.

**Reviewer #2**

**Comment:** This paper concerns “Characterization of nonlinear heart rate variability of end-stage renal disease patients after kidney transplantation”
It is a struggle for me to read the paper because of many language issues and a huge number of abbreviations. I am not familiar with all these cardiology terms and abbreviations, so that does not help. Although the subject is interesting, after trying to read it several times, at present I cannot comment properly because of the numerous textual issues.
Table 2 abbreviations after normal text would make it more readable.
A list of abbreviations is also required
A clear legend of the figures is necessary

The following sentences need improvement.
1. However, it is unknown whether this response is recovered after definitive treatment with kidney transplantation.
2. We studied HRV dynamics by obtaining short-term ECG recordings from kidney transplant recipients that underwent active standing stimulation
3. Larger estimated glomerular filtration rate (eGFR) in significantly correlated with
broader gross HRV in supine position and during active standing. The loss in HRV during active standing may indicate a preserved sympathetic response to external stimuli
4. is known that chronic sympathetic hyperactivity produces decreased HRV modulation with predominance of lowfrequency oscillations, and a blunted HRV response to active standing
5. This tool allows to graphically represent the recurrence of a system to a particular dynamical state
6. This maneuver allows studying of the effects of volume redistribution and sympathetic
stimulation of the cardiovascular system
7. However a kidney transplant is the treatment of choice for ESRD
8. We included 16 patients with ESRD after kidney transplantation that were recruited during followup consultation in a second-level hospital in Mexico with a median age of 32 [27-35] years old
9. and triple immunosuppression treatment with a calcineurin inhibitor plus prednisone and mycophenolate.
10. Subjects were asked to avoid caffeinated beverages and other stimulants
11. We name the final HRV time series as NN
12. We calculate the mean of NN time series (meanNN
13. Embedding dimension was calculated at the first local minimum reaching zero in the fixed amount of neighbors function
14. Categorical variables are reported as absolute frequency (relative frequency) and continuous
variables as median (interquartile range).
15. Time domain indices, meanNN, SDNN, SDSD, and pNN20 are larger during supine position in comparison to active standing
16. Few correlations are observed in supine position (SDSD, pNN20, DET and ENT), but during active standing a significant correlation is found between eGFR and most of HRVmeasures (SDNN, SDSD, Pnn20, LF, HF, LF/HF, DET, LAM, TT and T2).
17. Also, a larger standard deviation of 5-minutes intervals in 24 hours (SDANN) of kidney transplant recipients without diabetes than those recipients without diabetes
18. Here, patients after kidney transplantation
19. However, we found here that in transplant recipients, these measures increase their value during active standing.
20. change in healthy subjects in response to active standing. given that the rapid changes in heart rate
21. is due to dysautonomia. This
22. other experimental scenarios [14, 18], which are not found in patients with ESRD but after HD treatment.
23. approach such be conducted
24. our study do not have
25. it is likely that the changes in RQA measures in active standing (e.g. DET, ENT, LAM and TT) may be a result of fluid overload
26. resistance [5, 25], these
27. As we show in this
28. is related to better eGFR
29. to a improve in HRV
30. to explore this relation and determine
31. in comparison with supine position
32. a consistent predominance of vertical structures (LAM and TT).
33. The larger HRV statistical dispersion,
34. by better renal functioning (eGFR).

**Response:** We thank the reviewer for the observations. In the revised version we added the meaning of abbreviations, clear legends to the figures, and a list of abbreviations, as requested. We also made corrections to the phrases that the reviewer highlighted, we show them below. Sentences with letter “a” correspond to the previous version; those with letter “b”, to the revised version.

1a. However, it is unknown whether this response is recovered after definitive treatment with kidney transplantation.

1b. However, it is unknown whether the response to active standing is recovered after definitive treatment with kidney transplantation (page 2).

2a. We studied HRV dynamics by obtaining short-term ECG recordings from kidney transplant recipients that underwent active standing stimulation

2b. We studied HRV dynamics by obtaining 5-minutes ECG recordings from kidney transplant recipients that underwent an active standing test (page 2).

3a. Larger estimated glomerular filtration rate (eGFR) in significantly correlated with
broader gross HRV in supine position and during active standing. The loss in HRV during active standing may indicate a preserved sympathetic response to external stimul

3b. A larger estimated glomerular filtration rate (eGFR) was significantly correlated with broader HRV in supine position and during active standing. The narrower HRV during active standing may indicate a recovered sympathetic response to external stimuli (page 2).

4a. is known that chronic sympathetic hyperactivity produces decreased HRV modulation with predominance of lowfrequency oscillations, and a blunted HRV response to active standing

4b. chronic sympathetic hyperactivity produces decreased HRV modulation with a predominance of low-frequency oscillations and a blunted HRV response to active standing (page 3).

5a. This tool allows to graphically represent the recurrence of a system to a particular dynamical state

5b. This tool graphically represents the recurrences of a system to a particular dynamical state (page 3).

6a. This maneuver allows studying of the effects of volume redistribution and sympathetic
stimulation of the cardiovascular system

6b. This maneuver allows studying the effects of blood volume redistribution and sympathetic stimulation of the cardiovascular system (page 3)

7a. However a kidney transplant is the treatment of choice for ESRD

7b. However, a kidney transplant is the treatment of choice for ESRD (page 3).

8a. We included 16 patients with ESRD after kidney transplantation that were recruited during followup consultation in a second-level hospital in Mexico with a median age of 32 [27-35] years old

8b. We included 16 patients with ESRD after kidney transplantation recruited during follow-up consultation in a second-level hospital in Mexico with a median age of 32 [27-35] years old (page 4).

9a. and triple immunosuppression treatment with a calcineurin inhibitor plus prednisone and mycophenolate.

9b. triple immunosuppression regimen with a calcineurin inhibitor, prednisone and mycophenolate (page 4).

10a. Subjects were asked to avoid caffeinated beverages and other stimulants

10b. During recruitment, subjects were asked to avoid caffeinated beverages and other stimulants 24 hours before the evaluation (page 4).

11a. We name the final HRV time series as NN

11b. We named the final HRV time series as NN intervals (page 6).

12a. We calculate the mean of NN time series (meanNN

12b. We calculated the mean of NN time series (meanNN [s]), page 6.

13a. Embedding dimension was calculated at the first local minimum reaching zero in the fixed amount of neighbors function

13b. Embedding delay was chosen at the average mutual information function's first local minimum for each time series. The embedding dimension was calculated at the false nearest neighbors function (page 6).

14a. Categorical variables are reported as absolute frequency (relative frequency) and continuous variables as median (interquartile range).

14b. We report categorical variables as absolute frequency (relative frequency), and continuous variables as median (interquartile range), page 7.

15a. Time domain indices, meanNN, SDNN, SDSD, and pNN20 are larger during supine position in comparison to active standing

15b. The values of time domain indices, meanNN, SDNN, SDSD, and pNN20 are larger during supine position than active standing (Figure 2), page 7.

16a. Few correlations are observed in supine position (SDSD, pNN20, DET and ENT), but during active standing a significant correlation is found between eGFR and most of HRVmeasures (SDNN, SDSD, Pnn20, LF, HF, LF/HF, DET, LAM, TT and T2).

16b. Few correlations are observed between eGFR and HRV indices (SDSD, pNN20, DET and ENT) in supine position. During active standing, significant correlations are found between eGFR and most of HRV measures (SDNN, SDSD, Pnn20, LF, HF, LF/HF, DET, LAM, TT and T2), page 9.

17a. Also, a larger standard deviation of 5-minutes intervals in 24 hours (SDANN) of kidney transplant recipients without diabetes than those recipients without diabetes

17b. Also, a larger standard deviation of 5-minutes intervals in 24 hours has been observed in kidney transplant recipients without diabetes than those recipients with diabetes (page 10).

18a. Here, patients after kidney transplantation

18b. In this study, patients after kidney transplantation (page 10).

19a. However, we found here that in transplant recipients, these measures increase their value during active standing.

19b. However, we found here that these measures increase their value during active standing in transplant recipients (page 10).

20a. change in healthy subjects in response to active standing. given that the rapid changes in heart rate

20b. change in healthy subjects in response to active standing, given that the rapid changes in heart rate (page 11).

21a. is due to dysautonomia. This

21b. It has been suggested that the lack of a significant response in ESRD is due to autonomic nervous system impairment (dysautonomia). This (page 11).

22a. other experimental scenarios [14, 18], which are not found in patients with ESRD but after HD treatment.

22b. other experimental scenarios [15, 18], in which no correlation is found in patients with ESRD before HD, but such correlation is recovered after HD treatment.

23a. approach such be conducted

23b. approach should be conducted (page 11).

24a. our study do not have

24b. our study does not have (page 11).

25a. it is likely that the changes in RQA measures in active standing (e.g. DET, ENT, LAM and TT) may be a result of fluid overload

25b. it is likely that the changes in RQA measures observed in active standing (e.g. DET, ENT, LAM and TT) may be the result of fluid overload (age 12)

26a. resistance [5, 25], these

26b. resistance [5, 26]. These (page 12)

27a. As we show in this

27b. In this (page 12).

28a. is related to better eGFR

28b. is related to higher eGFR (page 12)

29a. to a improve in HRV

29b. a improvement in HRV (page 12)

30a. to explore this relation and determine

30b. to explore this relationship and to determine (page 12)

31a. in comparison with supine position

31b. compared with the supine position (page 13).

32a. a consistent predominance of vertical structures (LAM and TT).

32b. with a predominance of vertical structures in recurrence plots (measured through LAM and TT), page 13.

33a. The larger HRV statistical dispersion,

33b. The wider HRV statistical dispersion (page 13)

34a. by better renal functioning (eGFR).

34b. by better glomerular filtration rate (page 13).

**Reviewer #3**

**Comment:** Authors conducted research concerning the autonomic response (changes of HRV) in acitve standing in patients post-kidney transplant. Differences in HRV reagibilty were historically compared with ESKD patients (on dialyis) without transplant. The small number (n=16), no direct statistical comparison to ESKD and the very young test population (age 32yr) stimulate doubts about general associations and the comparability to ESKD overall. However the methodology of HRV measurements is sound, the Ms. well written and undstandable. The reviewer recommends that these consideration, in particular the very young population should be mentioned and more critically discussed in the paper.

**Response:** We thank the reviewer for the observations. In the revised version of the manuscript, we highlight that we cannot directly measure of HRV change due to kidney transplantation because we do not make direct comparisons between the HRV of subjects before and after kidney transplantation; we rather focus on describing the HRV response to active standing in this set of patients (Discussion section, page 10). We also stress the small sample size of this study and the uncharacteristic young age in ESRD patients who are transplant recipients (Discussion section, page 11).

**Reviewer #4**

**Comment:** The article focuses on the HRV (heart rate variability) of patients after kidney transplantation. Impaired HRV in patients with ESRD is known to be improved by HD. The improvement of the sympathetic stimulation mediated HRV response in active standing after kidney transplantation is still unknown and the subject of the present study. The study question is very interesting and concisely explained in the introduction.
The question was investigated using frequency-based metrics, RIQA (recurrence quantitative analysis) and short term ECG. The methodology is explained conclusively and is well suited to the investigation of the question.
It could be shown significantly that HRV is lower in active standing in kidney transplant patients than in supine. Significant differences in various HRV indices were shown in active standing. However, the article points out that other studies in patients with ESRD, not hemodialysis patients, showed no significant difference in HRV in active standing. The results are explained conclusively.
A disadvantage is the small number of subjects (16). There is also a lack of data (HRV) on the same patients before transplantation, as already noted by the authors

**Response:** We thank the reviewer for the observations. In the revised version of the manuscript, we highlight that we cannot directly measure of HRV change due to kidney transplantation because we do not make direct comparisons between the HRV of subjects before and after kidney transplantation; we rather focus on describing the HRV response to active standing in this set of patients (Discussion section, page 10). We also stress the small sample size of this study and the uncharacteristic young age in ESRD patients who are transplant recipients (Discussion section, page 11).
